# Parthenolide prevents resistance of MDA-MB231 cells to doxorubicin and mitoxantrone: the role of Nrf2

**DOI:** 10.1038/cddiscovery.2017.78

**Published:** 2017-12-04

**Authors:** Daniela Carlisi, Anna De Blasio, Rosa Drago-Ferrante, Riccardo Di Fiore, Giuseppina Buttitta, Marco Morreale, Christian Scerri, Renza Vento, Giovanni Tesoriere

**Affiliations:** 1 Laboratory of Biochemistry, Department of Experimental Biomedicine and Clinical Neurosciences (BioNec), University of Palermo, Polyclinic, Palermo, Italy; 2 Associazione Siciliana per la Lotta contro i Tumori (ASLOT), Palermo, Italy; 3 Laboratory of Biochemistry, Department of Biological, Chemical and Pharmaceutical Sciences and Technologies (STEBICEF), University of Palermo, Polyclinic, Palermo, Italy; 4 Sbarro Institute for Cancer Research and Molecular Medicine, Center for Biotechnology, Temple University, Philadelphia, PA, USA; 5 Faculty of Medicine and Surgery, Department of Physiology and Biochemistry, University of Malta, Msida, MSD, Malta; 6 Department of Pathology, Mater Dei Hospital, Msida, MSD, Malta

## Abstract

Triple-negative breast cancer is a group of aggressive cancers with poor prognosis owing to chemoresistance, recurrence and metastasis. New strategies are required that could reduce chemoresistance and increases the effectiveness of chemotherapy. The results presented in this paper, showing that parthenolide (PN) prevents drug resistance in MDA-MB231 cells, represent a contribution to one of these possible strategies. MDA-MB231 cells, the most studied line of TNBC cells, were submitted to selection treatment with mitoxantrone (Mitox) and doxorubicin (DOX). The presence of resistant cells was confirmed through the measurement of the resistance index. Cells submitted to this treatment exhibited a remarkable increment of NF-E2-related factor 2 (Nrf2) level, which was accompanied by upregulation of catalase, MnSOD, HSP70, Bcl-2 and P-glycoprotein. Moreover, as a consequence of overexpression of Nrf2 and correlated proteins, drug-treated cells exhibited a much lower ability than parental cells to generate ROS in response to a suitable stimulation. The addition of PN (2.0 *μ*M) to Mitox and DOX, over the total selection time, prevented both the induction of resistance and the overexpression of Nrf2 and correlated proteins, whereas the cells showed a good ability to generate ROS in response to adequate stimulation. To demonstrate that Nrf2 exerted a crucial role in the induction of resistance, the cells were transiently transfected with a specific small interfering RNA for Nrf2. Similarly to the effects induced by PN, downregulation of Nrf2 was accompanied by reductions in the levels of catalase, MnSOD, HSP70 and Bcl-2, prevention of chemoresistance and increased ability to generate ROS under stimulation. In conclusion, our results show that PN inhibited the development of the resistance toward Mitox and DOX, and suggest that these effects were correlated with the prevention of the overexpression of Nrf2 and its target proteins, which occurred in the cells submitted to drug treatment.

## Introduction

Breast cancer is the most frequent type of invasive malignancy in female worldwide.^1,2^ Triple-negative breast cancer (TNBC) is a very heterogeneous group of cancers, which includes forms lacking in the expression of estrogen receptor, progesterone receptor and human epidermal growth factor receptor 2 (HER 2). TNBCs, which account for 12–17% of all breast cancers,^3^ prevalently affect young women and are three times more frequent in African Americans than in Caucasian women.^4^ As TNBCs are not sensitive to endocrine therapy and HER 2 targeted therapy, standard treatment is represented by surgery with radiotherapy and adjuvant chemotherapy. TNBCs are very sensitive to chemotherapy during the first phase of treatment,^5^ successively the onset of chemoresistance leads TNBC cells to elude the cytotoxic effects of the drugs, developing a new relapsed form of the disease, often complicated by the production of metastasis.^6^ Novel therapy strategies are investigated^5^ to improve prognosis for TNBCs.

Doxorubicin (DOX) and Mitoxantrone (Mitox) are two antineoplastic drugs employed in the therapy of breast cancer. Both the drugs are multiring planar molecules that intercalate with DNA and inhibit the activity of topoisomerase II. DOX,^7,8^ which is considered the most active agent in breast cancer therapy, is an anthracycline antibiotic, whereas Mitox^9,10^ is a synthetic agent, belonging to the class of anthracenediones. Treatment with DOX or Mitox induces in most patients, after a positive response, the development of chemoresistance, associated with recurrence of the tumour form. The effectiveness of DOX is invalidated by cardiotoxicity,^11,12^ which represents a serious problem when, owing to the appearance of resistance against DOX, the patient is treated with repeated and increasing doses of this drug.

Being a multifactorial phenomenon, chemoresistance has a range of causes, which includes the following:^13–15^ activity of ATP-binding cassette (ABC) transporters, such as P-glycoprotein^16^ (MDR1 and ABCB1) and ABCG2, which function as drug efflux pumps and contrast the intracellular drug accumulation; activity of the pro-survival members of Bcl-2 family;^17^ alteration of drug target,^18^ as a consequence of mutation of some involved gene; epigenetic modifications of genes;^19^ and finally acquisition of resistance to apoptosis,^20^ due to downregulation of one or more factors. Moreover, many factors and signalling pathways are involved in chemoresistance. In particular, aldehyde dehydrogenase,^21^ myc,^22^ Akt/PKB^23^ and Wnt/*β*-catenin^24^ pathways exert important roles in specific cases. With regard to TNBC cells, chemoresistance has been attributed to senescent-like cells induced by therapeutic treatment.^5^ This event has been interpreted as a strategy of cells to avoid the apoptotic process.

The transcription factor NF-E2-related factor 2 (Nrf2), which regulates the expression of a battery of genes and is involved in antioxidant and cytoprotective responses and in ATP-dependent drug efflux pumps, is thought to have a crucial role in the control of redox homeostasis and the cell protection against chemical and radiation stress.^25–27^ Therefore, Nrf2 overexpression in tumour cells favours development of chemoresistance. Consequently, chemical inhibitors of Nrf2 can represent a new tool to prevent the resistance induced in tumour cells by chemotherapeutic agents.

Parthenolide (PN), a sesquiterpene lactone found in *Tanacetum parthenium*, known for its anti-inflammatory activity, exerts anti-tumour effects on many cancer cells, whereas it is ineffective in normal cells.^28,29^ Previously, we have shown that PN induced cytotoxicity in MDA-MB231 cells,^30^ which are the most studied TNBC cells, through the stimulation of oxidative stress and autophagy. Moreover DMAPT, a soluble analogue of PN, markedly decreased tumour growth in mice bearing xenografts of MDA-MB231 cells and enhanced survival of treated mice.^30^ More recently,^31^ we produced mammospheres from three distinct lines of TNBCs and showed that both PN and DMAPT suppressed production of spheres and induced cytotoxicity in stem-like cells derived from their dissociation.

In this study we submitted MDA-MB231 cells to selection treatment with DOX and Mitox, in order to produce resistant cells. PN prevented the development of chemoresistance and the overexpression of Nrf2 and correlated proteins found in selected cells.

## Results

### PN increased the cytotoxic effect of Mitox and DOX in MDA-MB231 cells

**Figure 1:**
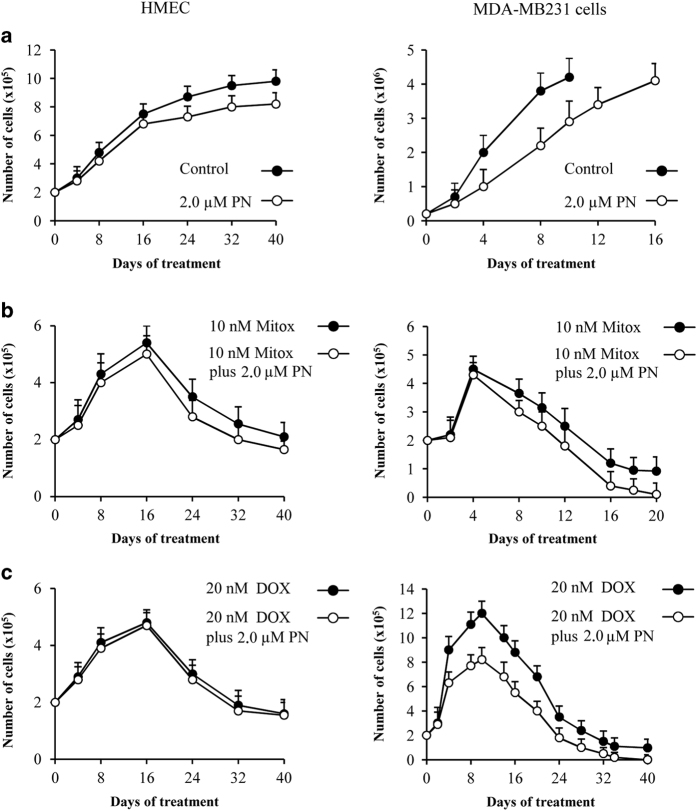
The effects exerted by Mitox and DOX on the growth of cultured HMEC and MDA-MB231 cells. The influence of PN. Cells (2×10^5^) were seeded in 25 cm^2^ flasks in 7.5 ml of medium. Viability was measured every 2 days by means of Trypan Blue assay. (**a**) HMEC and MDA-MB 231 cells cultured in medium in the absence or the presence of 2.0 *μ*M PN. Growth of untreated MDA-MB 231 cells was stopped at 10th day when the cultures were near to the confluence, whereas in the presence of PN was stopped at 16th day. (**b**) Cells cultured in medium containing 10 nM Mitox without or with 2.0 *μ*M PN. (**c**) Cells cultured in medium containing 20 nM DOX without or with 2.0 *μ*M PN. All the values are the means±S.D. of four different experiments.

Figure 1a shows the time course of the growth of cultured MDA-MB231 cells compared with normal human mammary epithelial cells (HMECs). Cell growth of the two lines increased progressively with time, with MDA-MB231 cells growing much more rapidly than HMEC. MDA-MB231 cells reached the confluence (4.2×10^6^ cells) at 10th day of culture, when the number of HMEC were only 5.8×10^5^ cells. When 2.0 *μ*M PN was added to the cultures a slower progression of the growth was observed in comparison with untreated cells (Figure 1a) with the difference being higher for MDA-BM231 cells than for HMEC.

Mitox and DOX exert a strong cytotoxic effect on breast cancer cells. The time courses of their effects on the viability of HMEC and MDA-MB231 cells are shown in Figure 1b (Mitox) and 1c (DOX). During the first phase of treatment with either Mitox (0–4 days) or DOX (0–10 days) MDA-MB231 cell number increased, although to a degree that was much lower than for untreated cells (Figure 1a). In the second phase, viability progressively decreased to very low values with the decrease on Mitox treatment being greater and more rapid than with DOX. Similarly, two phases were also observed for HMEC, but the decrease during the second phase was less rapid and less evident than with MDA-MB231 cells. For instance, treatment with Mitox reduced MDA-MB231 cells to only 1.0×10^5^ at the 20th day, whereas HMEC were reduced to 4.4×10^5^. PN (2.0 *μ*M) increased toxicity of both Mitox and DOX, so that at the 20th day of treatment with Mitox and 40–42th day with DOX viable cells were not identified any more in the cultures, whereas when the cells were treated with the drugs alone without PN a small amount of cells remained viable in the last phase of treatment. We hypothesized that this difference was determined by development of drug resistance and that PN prevented this event.

Interestingly, during Mitox treatment monstrous, senescent-like cells appeared by the 9th day of treatment and by the 20th day reached about 30% of the total cells, whereas in cells treated with DOX senescent-like cells appeared only by the 20th day. The addition of PN did not substantially modify production of these atypical cells.

Finally, the observation that the addition of PN to the drugs did not cause significant effects on HMEC (Figures 1b and c) confirmed the limited activity of PN on normal cells.

### PN inhibited the development of resistance induced by Mitox and DOX

**Figure 2:**
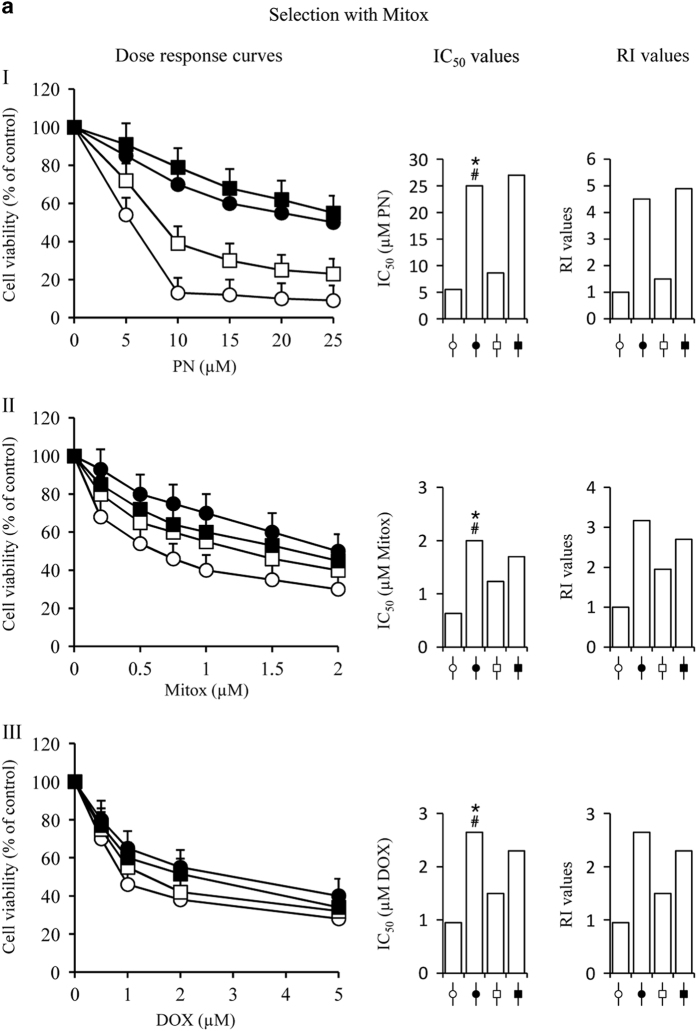

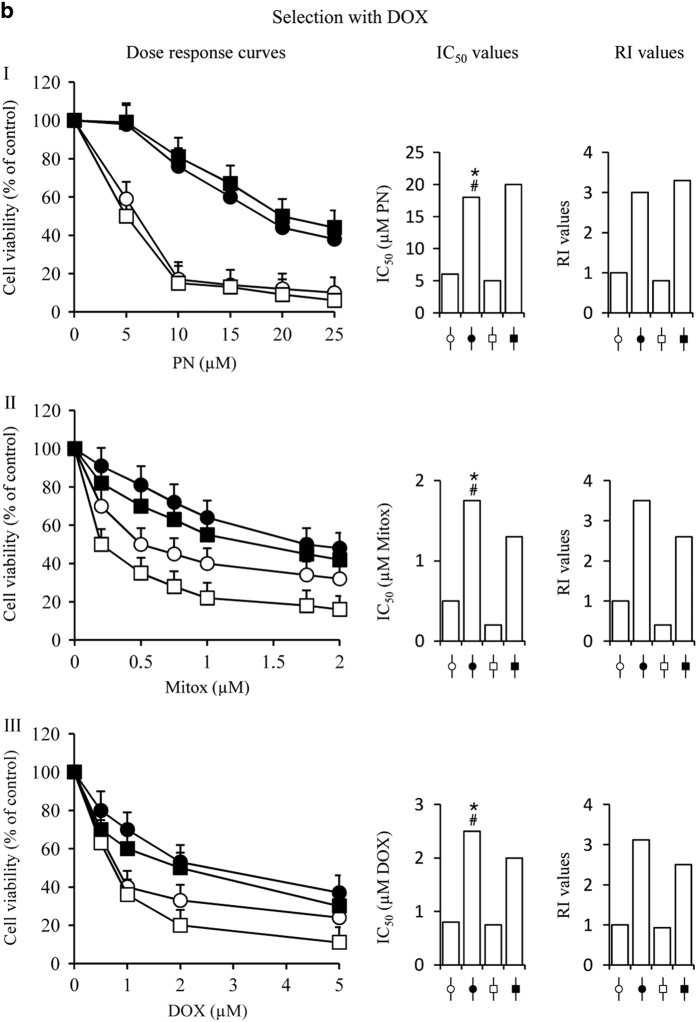
MDA-MB231 cells developed resistance under the exposure to Mitox or DOX. PN partially prevented this event. MDA-MB231 cells (5×10^5^) were seeded in 75 cm^2^ flasks in 15.0 ml of medium. Selection with Mitox and DOX was performed as reported in methods. In order to investigate whether PN prevents the development of resistance, cells were pre-treated for 2 days with 2.0 *μ*M PN alone, then the drugs were added and the combined treatment was protracted for the total selection time. At the end cells of all the conditions were maintained for other 5 days in medium without both drugs and PN. Panels **a** and **b** report results concerning cells submitted to selection with Mitox or DOX, respectively. In order to ascertain the degree of resistance reached in the various conditions, the cells were collected and employed, as reported in methods, to test the effect on viability exerted for 48 h by various doses of (I) PN, (II) Mitox and (III) DOX. Viability was ascertained for the following conditions: parental cells (--○--); cells treated with drugs to acquire resistance (--●--); cells treated with drugs plus 2.0 *μ*M PN (--□--); cells treated with drugs plus PN as in the previous condition, but with the difference that 2.0 mM NAC were added in the samples performed to test viability (--■---). Viability was assessed by MTT assay and reported for each condition as percentage of cells treated with vehicle only. For each compound tested and for each condition the Figures report dose–response curve, IC_50_ value calculated from dose–response curves and the RI. The results are the means of four different experiments±S.D. *P<0.01 *versus* parental cells; #*P*<0.01 *versus* cells treated with Mitox or DOX plus PN.

As reported in Materials and Methods, MDA-MB231 cells were submitted to treatment with Mitox or DOX for a total selection time of 25 days, in order to produce resistant cells. At the end, selected cells were employed to evaluate the effects of various doses of Mitox, DOX and PN on their viability. To ascertain the acquisition of resistance, IC_50_ values were at first calculated from dose–response curves, then the resistance indices (RIs) were obtained by evaluating the ratio between the corresponding IC_50_ and the IC_50_ measured for parental cells, for each case of drug treatment. Figures 2a and b show dose–response curve, IC_50_ and RI values for the three compounds and for each cell condition. The results demonstrate that the treatment produced resistant cells, as represented by an increase of RIs to 4.5, 3.1 and 2.7 values with Mitox-treated cells and to 3.0, 3.5 and 3.1 values with DOX-treated cells, depending if the resistance was evaluated toward PN, Mitox or DOX, respectively.

It is known that PN at high doses (10–20 *μ*M) exerted cytotoxic effects on MDA-MB231 cells by stimulating ROS generation,^30^ but was unable to induce resistance in these cells. However, when PN was added to Mitox or DOX, over the total selection time it prevented the development of resistance. In particular, PN caused a partial, but considerable effect, when it was added to Mitox, as shown by IR values (Figure 2a), whereas it completely prevented resistance when added to DOX (Figure 2b).

This preventive effect of PN was clearly observed at a concentration equal or superior to 2.0 *μ*M. This dose was chosen for our experiments, because it did not induce toxic effects on cultured cells, when administered alone.

Moreover, PN was added to the samples 2 days before the drugs, as this resulted in a higher effect on the prevention of resistance than when PN was added together with the drugs. These results suggest that PN induced molecular changes in the cells, which opposed the development of resistance.

The inhibitory effect of PN on development of resistance was contrasted by the addition of 2.0 mM NAC to the samples utilized to study dose–response curves. In particular, the PN effect was abolished by NAC when the resistance was ascertained towards the same PN and markedly reduced when resistance was ascertained towards Mitox and DOX (Figures 2a and b). The effect exerted by NAC suggested that PN prevented the development of resistance inducing changes, which favour ROS generation.

### Nrf2 and correlated proteins were upregulated in resistant cells, an effect which was partially prevented by PN

Through western blotting analysis on whole-cell lysates, it was shown that the development of resistance in MDA-MB231 cells was accompanied by overexpression of some factors correlated with antioxidant response or cell survival. At first, we evaluated the expression of Nrf2,^27^ a critical regulator of antioxidant activities. Then our study was extended to MnSOD and catalase, two activities which control the intracellular level of ROS;^32,33^ HSP70,^34^ a heat shock protein which inhibits apoptosis and induces drug resistance; P-gp,^35^ a transporter that controls the efflux of anticancer drugs and finally Bcl-2,^36^ a fundamental antiapoptotic protein.

**Figure 3:**
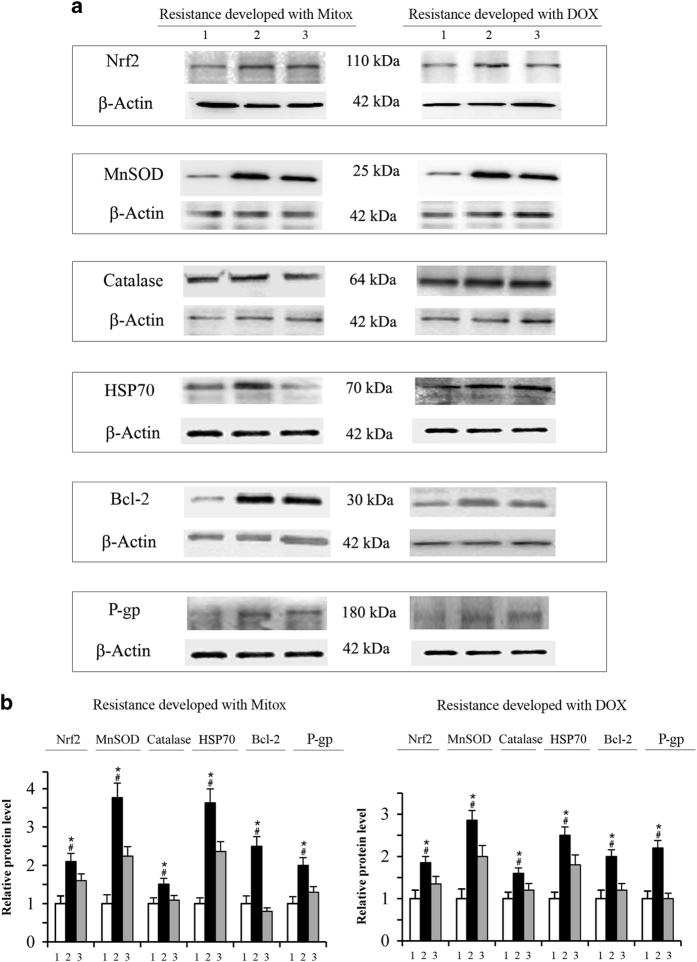
Development of resistance induced overexpression of Nrf2 and correlated proteins, an effect which was partially prevented by PN. MDA-MB231 cells were submitted to treatment with Mitox or DOX, as reported in methods, in order to develop the resistance. In some samples 2.0 *μ*M PN were added two days before the drugs and maintained for the total selection time together with the drugs. Western blotting analysis was performed as reported in methods. (1) Parental cells; (2) cells selected with Mitox or DOX; (3) cells treated with Mitox or DOX plus PN. (**a**) Images of western blotting analysis. Results are representative of four different experiments. (**b**) Estimation by densitometric analysis of the intensity of the bands. The results are the means of four distinct experiments±S.D. *P<0.01 *versus* parental cells; #*P*<0.01 *versus* cells treated with Mitox or DOX plus PN.

Nrf2 level was found to be enhanced by 110 and 85% in cells selected with Mitox and with DOX, respectively, in comparison with parental cells (Figures 3a and b). Moreover, western blotting analysis demonstrated that the nuclear content of Nrf2 with respect to the total content of the protein increased from 40% in parental cells to 75% in both the selected cells (not shown). In addition, the level of the other five proteins examined, namely MnSOD, catalase, HSP70, Bcl-2 and P-gp, were markedly increased in selected cells (Figure 3a). In particular, the increments for Mitox-selected cells ranged from 50% for catalase to 270% for MnSOD, whereas for DOX-selected cells the variation was from 60% for catalase to 180% for MnSOD (Figure 3b).

Previously^31^ we showed that PN downregulated Nrf2 expression in stem-like cells derived from MDA-MB 231 cells. This finding suggested to us that PN may contrast the increased expression of Nrf2 in selected cells. In accordance, our results showed that when 2.0 *μ*M PN was added in association with the drugs over the total selection time, the increments of both Nrf2 and correlated factors were significantly reduced both in Mitox- and DOX-selected cells. In particular, co-treatment reduced by about 50% the increment of Nrf2 expression in both selected cells (Figures 3a and b). For the other proteins the reduction was always higher than 40% and in some cases (Bcl-2 and P-gp) the increments were completely suppressed. Finally, the relative level of nuclear Nrf2 was also decreased in cells submitted to combined treatment to 40–45% of the total content (not shown).

### Cell ability to generate ROS was inhibited in resistant cells. Also, this effect was partially prevented by PN

**Figure 4:**
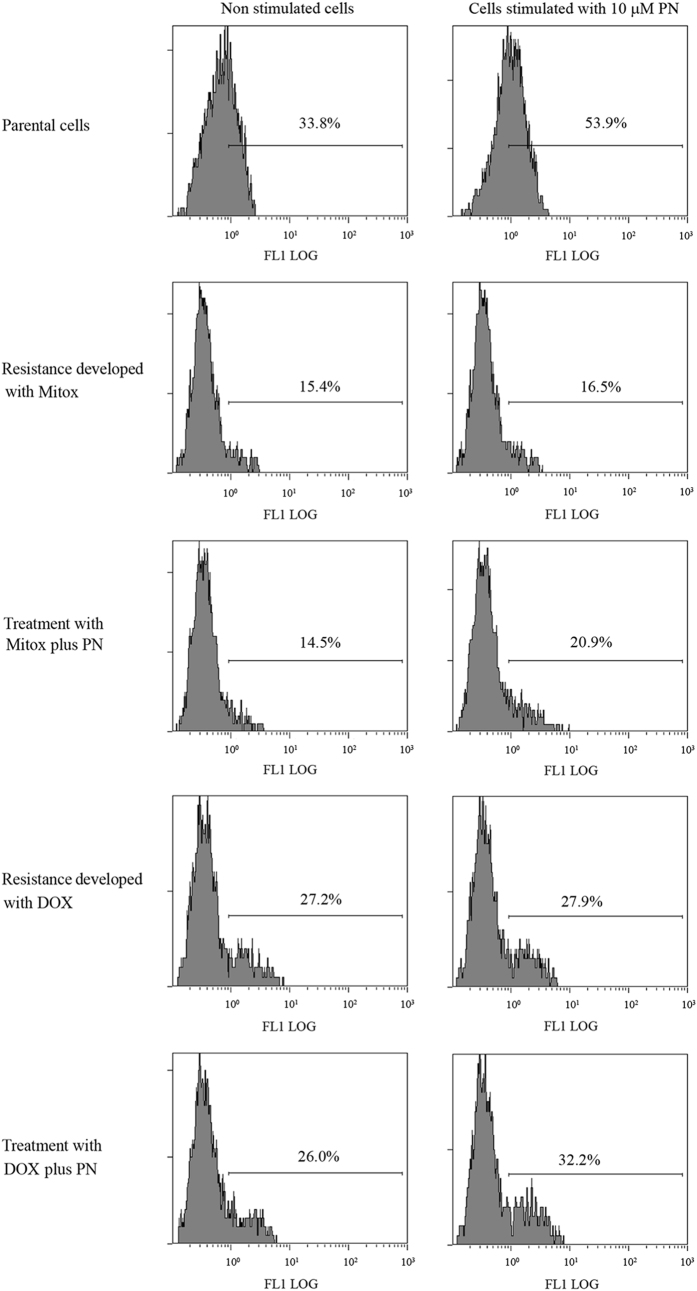
The development of resistance inhibited ROS generation. Addition of 2.0 *μ*M PN to the drugs during the selection time reduced the inhibitory effect on ROS generation. Parental and resistant cells were treated for 1 h with 10 *μ*M PN in order to stimulate production of ROS. At the end 10 *μ*M H2-DCFDA were added for 20 min, then the cells were collected and employed for cytofluorimetric analysis. Results are representative of three different experiments.

We evaluated in some experiments the ability of selected cells, in comparison with parental cells, to produce ROS in response to a suitable stimulation. As PN, as previously shown,^30^ is a valid inductor of ROS in MDA-MB231 cells, in these experiments the cells were stimulated for 1 h with 10 *μ*M PN and then submitted to DCF test,^37^ using cytofluorimetric analysis. Parental cells are particularly able to generate ROS, as DCF-positive cells increased from 33.8% in non-stimulated cells (basal level) to 53.9% after stimulation (Figure 4). Interestingly, the basal level of DCF-positive cells fell from 33.8% in parental cells to 15.4% in Mitox-selected cells and to 27.2% in DOX-selected cells. Similar decrements were observed also when the cells were treated over the selection time with the drugs plus PN. These results suggested that low levels of intracellular ROS were present in the surviving cells at the end of the selection time. It is interesting to note that the amount of DCF-positive cells was not significantly modified by stimulation neither in Mitox- nor in DOX-selected cells. Therefore, we concluded that selected cells were unable to produce ROS, even when submitted to a suitable stimulation. Instead, cells treated during the selection time with drugs plus PN were capable of generating ROS, as indicated by the significant responses following adequate stimulation. In particular DCF-positive cells increased from 14.5% to 20.9% (*P* <0.01) in cells treated with Mitox plus PN and from 26.0% to 32.2% (*P* <0.01) in cells treated with DOX plus PN.

### Knockdown of Nrf2 decreased the expression of target activities and prevented development of drug resistance

**Figure 5:**
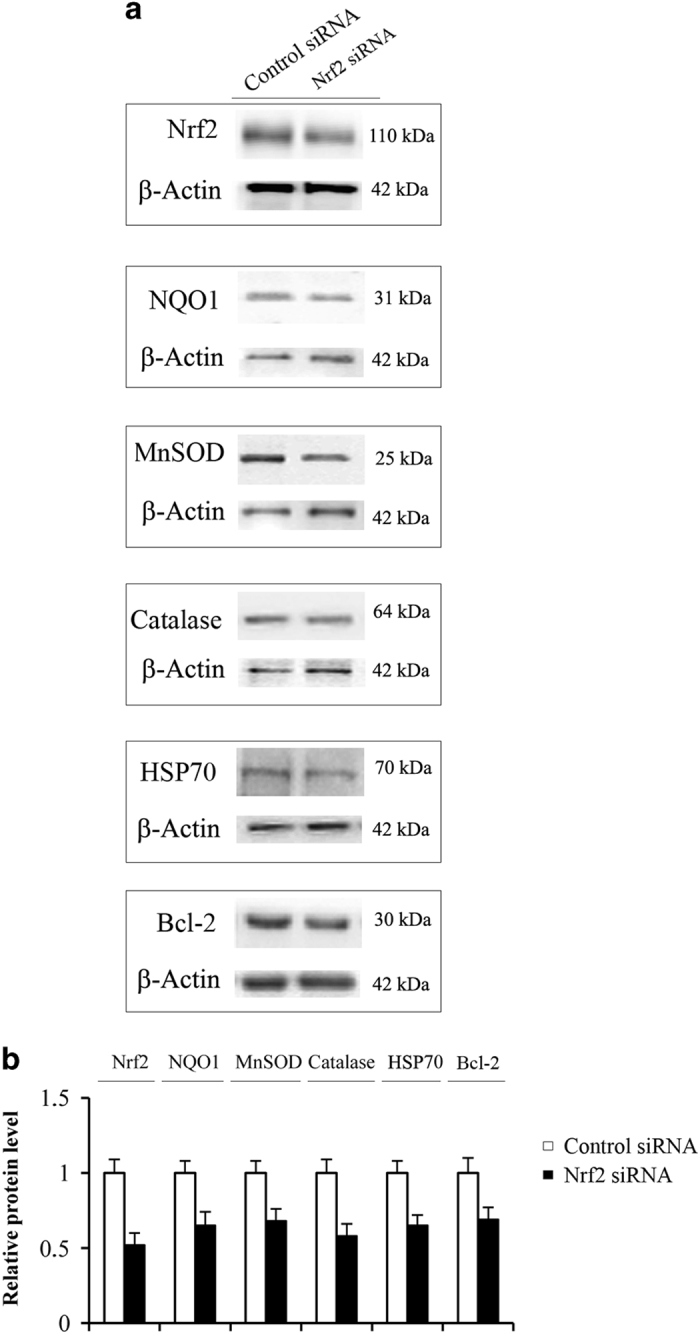
Silencing of Nrf2 decreased the expression of antioxidant and cytoprotective proteins. Nrf2 was silenced in parental MDA-MB231 cells, as reported in methods. After 24 h whole cellular extracts were prepared and submitted to western blotting analysis. (**a**) Images of western blotting analysis. Results are representative of four different experiments. (**b**) Estimation by densitometric analysis of the intensity of the bands. The results are the means of four distinct experiments±S.D.

It is known that Nrf2 controls transcriptional activity of many antioxidant and detoxifying genes. Moreover, high levels of Nrf2 were found in tumour cells resistant to chemotherapeutic drugs.^25^ This finding was confirmed by the present paper, showing that acquisition of resistance was accompanied by enhancement of Nrf2 expression. In addition, brusatol,^38^ a compound that decreases Nrf2 level, sensitized tumour cells to the drugs. Therefore, we hypothesized that PN can exert its effect against acquisition of drug resistance by preventing the increment of Nrf2 expression, which occurs during the selection time, and consequently also those of the correlated antioxidant and protective factors. In order to demonstrate in MDA-MB231 cells the relationships between Nrf2 and antioxidant activities, as well as between Nrf2 and the acquisition of resistance, we silenced Nrf2 expression in parental MDA-MB231 cells by using Nrf2 small interfering RNA (siRNA). Western blotting analysis showed (Figure 5) that, after 24 h from transfection, the level of total Nrf2 was strongly diminished in cells transfected with Nrf2 siRNA in comparison with cells treated with control siRNA. In addition, the relative level of nuclear Nrf2 decreased by 50% compared with control (not shown). Significant decrements (30–40%) were also observed for the intracellular levels of NQO1, HSP70, MnSOD catalase and Bcl-2 (Figure 5). These results demonstrated that these activities are under Nrf2 control in MDA-MB231 cells.

In other experiments, parental MDA-MB231 cells were at first transfected with Nrf2 siRNA and then treated for 7 days with 10 nM Mitox. As shown in Table 1, in these cells IC_50_ values and consequently the RIs, measured towards the three selected compounds (PN, Mitox and DOX), were much lower than the values ascertained for cells submitted to the identical treatment with Mitox, but after transfection with control siRNA. These results demonstrated that down-regulation of Nrf2 and correlated proteins partially prevented acquisition of resistance in cells treated with Mitox.

**Figure 6:**
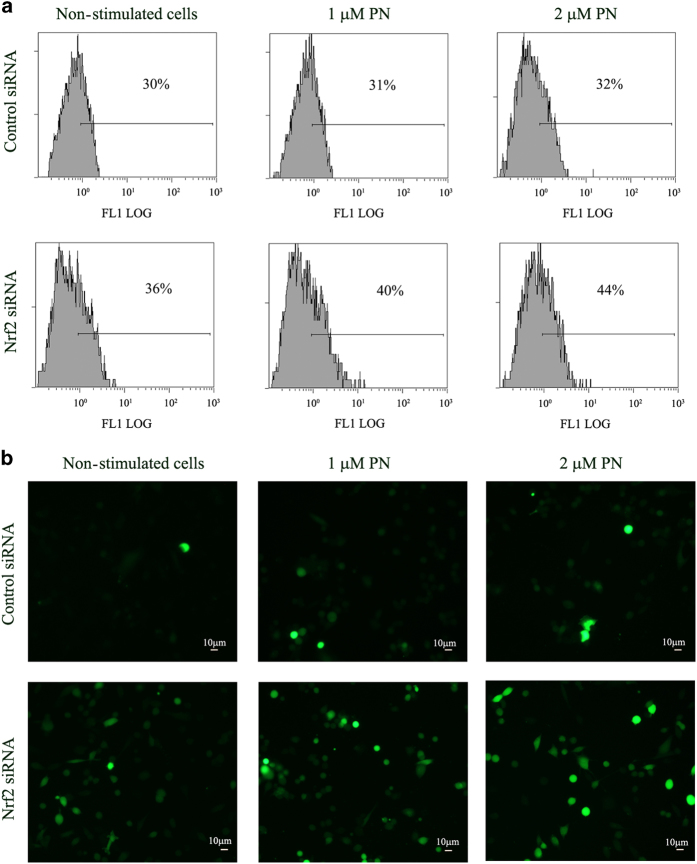
Silencing of Nrf2 in parental MDA-MB231 cells enhanced the intracellular level of ROS and the effect of PN on ROS generation. Nrf2 was silenced in parental MDA-MB231 cells as reported in methods. After 5 h of transfection the cells were employed, as described in methods, for (**a**) cytofluorimetric analysis and (**b**) visualization by fluorescence microscope. Results are representative of three different experiments.

As Nrf2 stimulates the expression of antioxidant activities, we hypothesized that the enhancement of Nrf2 can be responsible for inhibition of ROS generation, whereas on the contrary downregulation of Nrf2 can favour production of ROS. To demonstrate the validity of this hypothesis, Nrf2-silenced cells were submitted to DCF test. Cytofluorimetric analysis, reported in Figure 6a, showed that in non-stimulated conditions the proportion of DCF-positive cells was higher in Nrf2-silenced cells than in cells transfected with control siRNA (control cells). Moreover, this proportion increased after stimulation of Nrf2-silenced cells for 1 h with low doses of PN (1-2 *μ*M), whereas a much minor effect was observed in control cells. However, at higher doses of PN the difference between the two conditions was no more evident. To confirm these results we followed another procedure, based on fluorescence microscopy. As shown in Figure 6b, in Nrf2-silenced cells fluorescent cells appeared already in non-stimulated conditions and its number increased after stimulation for 1 h with 1–2 *μ*M PN, whereas in the control fluorescent cells were not visible before stimulation and appeared in a modest number after stimulation with low doses of PN.

These results, taken together, showed that silencing of Nrf2 in MDA-MB231 cells enhanced cells ability to generate ROS, in accordance with the decrement of the expression of the antioxidant and cytoprotective activities observed in the same silenced cells. These results are in agreement with the effect induced by PN, when it was added to the drugs during the selection time. In fact, PN in this case contrasted the overexpression of Nrf2 and at the same time preserved cell ability to generate ROS.

## Discussion

It is known that low levels of intracellular ROS assure survival and viability of both resistant^39^ and stem cells.^40,41^ In both cases, the low level of ROS seems to be correlated with upregulation of antioxidant activities. Instead high levels of ROS are harmful to the cells favouring DNA damage and cell death by apoptosis or necrosis.^42^ It is reasonable to suppose that in order to maintain ROS generation at low levels, cells increased Nrf2 expression. The activity of Nrf2 is under the control of Keap1,^43–45^ a factor which interacts in the cytoplasm with Nrf2, inducing its ubiquitination and proteasomic degradation. Various stress conditions lead to the modification of a cysteine residue in Keap1, causing Nrf2 release and its consequent translocation into the nucleus, where it induces transcriptional activity of genes bearing the antioxidant response element (ARE). Thus, stress conditions, through Keap1, control cellular redox homeostasis. Accumulating evidence suggests that by stimulating a wide range of genes, Nrf2 exerts a crucial role in the protection against oxidative stress, in detoxification of drugs and cytoprotective events.^45^ Among the enzymes involved in the protection against oxidative stress, MnSOD and catalase^32^ control the intracellular level of ROS, with MnSOD converting superoxide anion to hydrogen peroxide, which is further converted into water by catalase. It is known that both the activities are under the control of Nrf2 (Kobayashi and Yamamoto^44^) and that their expression markedly increases after Nrf2 stabilization by D3T.^46^ In addition, Nrf2 intervenes in the upregulation of P-gp,^47^ a member of ABC transporter family, that favours the efflux of anthracyclines and taxane.^35^ Moreover, a closed relationship exists between Nrf2 and HSP70, as sulfhydryl-reactive inducers of the Keap1/Nrf2/ARE pathway upregulate HSP70.^48^ HSP70 is a well-known family of the chaperone proteins HSPs,^49–51^ which are enhanced by cellular stress, such as inflammation, high temperature and oxidative stress, and are involved in the folding of synthetized polypeptide and in the interaction with apoptotic factors and consequently in their inhibition. Owing to this last ability, HSP70 family supports the survival of cells and thus favours drug resistance. Finally, Nrf2 exerts a cytoprotective effect also by inducing the transcription of Bcl-2 and Bcl-XL, two fundamental inhibitors of apoptosis.^52,53^

There is evidence that cell resistance is correlated with Nrf2 expression.^54^ In particular, it is known that Nrf2 induced radioresistance.^55^ Moreover, Nrf2 overexpression enhanced resistance of cultured cells to cisplatin, DOX and etoposide.^25^ Accordingly, also resistance to tamoxifen in breast cancer patients was correlated with increased expression of both Nrf2 and other antioxidant proteins.^56^ Inversely, downregulation of Nrf2, caused by transfection of Nrf2 siRNA^26^ or by the inhibitor brusatol,^38^ enhanced drug susceptibility of tumour cells. Similarly, epigallocatechin-3-gallate,^57^ an effective inhibitor of Nrf2, efficaciously prevented resistance against tamoxifen. In conclusion, drug resistance, high expression of Nrf2, and correlated proteins and low ability to generate ROS are three aspects closely linked each other.

To improve the effectiveness of chemotherapeutic treatment of TNBC forms we chose to associate PN to the drugs, in order to increase their activity by preventing development of drug resistance. This choice was suggested by the observation^58^ that PN enhanced DOX cytotoxicity in human lung cancer cells. Moreover, our previous study on the cytotoxic effects induced by PN on stem-like cells, derived from MDA-MB231 cells,^31^ suggested that PN can prevent drug resistance by decreasing Nrf2. To ascertain the role of PN we submitted MDA-MB231 cells to selection treatment with Mitox or DOX either in the absence or in the presence of 2.0 *μ*M PN. In the absence of PN, exposure to the drugs increased IC_50_, reaching values about threefold higher than in parental cells and in addition caused overexpression of Nrf2 protein. This effect was associated with the increase of some proteins, such as MnSOD, catalase, HSP70, Bcl-2 and P-gp, which are upregulated by Keap 1/Nrf2/ARE pathway and exert important roles as antioxidant and cytoprotective activities. Finally, in these cells the ability to generate ROS was particularly diminished, in accordance with the high expression of antioxidant activities.

When PN was added to Mitox or DOX, over the total selection time required to induce resistance, it partially prevented Nrf2 overexpression, as well as diminished the levels of the correlated proteins. Owing to the effect on MnSOD and catalase, the antioxidant power decreased and the cells maintained the ability to generate ROS under an adequate stimulus. The concomitant decrease in the levels of the cytoprotective agent HSP70 (Bozaykut *et al.*^49^) and the anti-apoptotic factor Bcl-2 (Zhou *et al.*^55^) contributed to reduce the defence mechanisms of the cells. Consequently, RI values decreased and the cells continued to die, whereas an amount of cells remained viable in the samples treated with the drugs alone without PN.

Our results strongly suggested that Nrf2 has a crucial role in the control of antioxidant power and in the fate of MDA-MB231 cells. In fact silencing of Nrf2 decreased resistance of cells and the levels of antioxidant proteins, whereas the ability to generate ROS increased. In conclusion, knockdown of Nrf2 caused similar effects to those ascertained when PN was added to the drugs during the selection treatment.

It is interesting to note that PN was unable to induce resistance, because chemoresistance is correlated with the increment of antioxidant activities and the reduction of the intracellular ROS level, whereas PN induces opposite effects. Instead, cells which acquired resistance through the exposure to drugs exhibited resistance also towards PN, because high levels of Nrf2 and antioxidant proteins prevented PN cytotoxicity, which is based on stimulation of ROS generation.

An important question is whether the increase in Nrf2 expression, which is typical of resistant cells, is stable or can be lost after a prolonged treatment with the same PN or other compounds capable of down-regulating Nrf2 expression. We shall elaborate further on this interesting aspect in a future work.

It has been shown that the promoter region of *Keap1* gene is hypermethylated in many lines of cancer cells, such as colorectal cancer^59^ and lung cancer.^60^ This hypermethylation is responsible for inactivation of Keap1 function with the concomitant activation of Nrf2, which is associated with cancer development and drug resistance.^61^ Genistein, a natural isoflavone, inhibits the activity and the expression of DNMT1.^62^ Owing to this property genistein decreases DNA methylation in the promoter region of tumour suppressor genes,^62^ as well as in that of *keap1* gene. As a consequence genistein increases the expression of Keap1 mRNA.^63^ This effect reduces Nrf2 transition to the nucleus with the consequent decrement of the antioxidant activities and increment of ROS level. By inhibiting DNMT1 activity, through the alkylation of cysteine 1226 in the catalytic domain of the enzyme, PN also causes global DNA hypomethylation.^28,64^ Therefore, we suggest that PN, like genistein, can decrease methylation in the promoter region of the *Keap1* gene and consequently can reduce Nrf2 level in the nucleus preventing the acquisition of drug resistance. It seems possible that the action of PN on Nrf2 can really consist in limiting, by means of a similar mechanism, the overexpression of this protein which occurs in particular cases, such as in resistant cells.

Chemoresistance leads to the survival of a heterogeneous population of tumour cells. A small fraction of these cells, named cancer stem cells (CSCs),^1,15,65^ can give rise to a new recurrent form of the tumour, because they are capable of self-renewal and differentiation. It is known that CSCs are resistant to therapy, because they exhibit one or more defence mechanisms. Therefore, after drug treatment the proportion of these cells increases. This favours the action of stimulatory agents, inducing the proliferation of CSCs and the production of a recurrent form of the tumour, thus mirroring the complexity and the cellular heterogeneity of the primary form. Previously^31^ we produced mammospheres from three lines of TNBC cells, including MDA-MB231 cells, and demonstrated that PN, through its cytotoxic effects on stem-like cells, prevented their production.

Our study demonstrates that PN prevented the acquisition of resistance induced by Mitox and DOX treatment in MDA-MB231 cells. This effect was mediated by inhibition of overexpression of both Nrf2 and its target activities. Therefore, within MDA-MB231 cell lines, PN not only exerts toxic effects on stem-like cells, which are responsible for tumour recurrence, but also prevents drug resistance. However, because PN exhibits a scarce bioavailability, we suggest that DMAPT, an analogue of PN, which shows the same mechanism of action of PN, but has enhanced bioavailability, can be considered in association with the drugs for a new therapeutic strategy for TNBCs.

## Materials and methods

### Chemicals and reagents

PN, Mitox, DOX and all chemicals, except when stated otherwise, were supplied by Sigma-Aldrich (Milan, Italy). Stock solution of PN was prepared in dimethylsulfoxide (DMSO) and then diluted to final concentration in the culture medium. DMSO employed as vehicle never exceeded 0.04% and had no discernible effects on cells in comparison with the control. Mitox and DOX were prepared in ddH2O and diluted to final concentration in the culture medium.

### Cell cultures

The human breast cancer cell line MDA-MB231 was purchased from Interlab Cell Line Collection (Genova, Italy) and grown as monolayer in DMEM culture medium, supplemented with 10% fetal calf serum, 2.0 mM glutamine, 1% non-essential amino acids. HMEC were purchased from Lonza (Walkersville, MD, USA) and grown according to the manufacturer’s instructions. Cells were grown in an incubator at 37 °C in a humidified atmosphere containing 5% CO_2_.

### Cell viability

Cell viability was ascertained by MTT colorimetric assay, as previously described.^30,31^

For the experiments reported in Figure 1, HMEC and MDA-MB231 cells (2×10^5^) were seeded in 25 cm^2^ flask in 7.5 ml of DMEM culture medium containing the drugs. Samples were evaluated every day by light microscopic observation. At established times, cultures were detached and an aliquot of cell suspension was diluted with 0.4% trypan blue and counted in a hemocytometer under a microscope at ×200 magnification.^66^ The dye is admitted by dead cells, which appear blue, whereas it is excluded by viable cells, which appear transparent. In our experiments the number of the transparent, viable cells was counted. The counting was repeated three fold for each condition and the mean of the three values was considered for the results.

### Generation of resistant cells

In order to produce drug resistant cells MDA-MB231 cells (5×10^5^) were seeded in 75 cm^2^ flask in 15 ml of medium. Two procedures were followed. Procedure I: cells were treated for 15 days with 5 nM Mitox and then for other 10 days with 10 nM Mitox. Procedure II: cells were treated for 15 days with 10 nM DOX and then for other 10 days with 20 nM DOX.

PN was employed in order to prevent the development of drug resistance. In this case, MDA-MB231 cells were pre-treated for 2 days with 2.0 *μ*M PN in 75 cm^2^ flask. Then the cells were collected, counted, seeded (5×10^5^ cells) in 75 cm^2^ flask in 15 ml of medium and treated following one of the above reported procedures. During this treatment, PN (2.0 *μ*M) was always maintained in the medium together with Mitox or DOX. After 25 days the cells of all the conditions remained for other 5 days in the medium without both drugs and PN. Finally the cells were collected and counted. In order to ascertain resistance towards Mitox, DOX and PN, cells (8×10^3^/well) were plated in 200 *μ*l of DMEM in a 96-well plate and treated for 48 h with various doses of the three compounds. Control samples were treated with vehicle only. Cell viability was evaluated by MTT assay. Dose–response curve permitted to calculate for every condition IC_50_, which corresponds to the dose capable of inhibiting cell proliferation by 50%. The degree of resistance was calculated by the RI, which corresponds for every condition to the ratio between the related IC_50_ and the IC_50_ calculated for the parental cells.

### Evaluation of ROS generation

Intracellular ROS were detected using the redox-sensitive fluorochrome H_2_-DCFDA (Molecular Probe, Life Technologies, Eugene, OR, USA). Cells (10^5^/well) were seeded in six-well plates and, after 24 h, were incubated with PN for 1 h. At the end the cells were washed with PBS and incubated with 10 *μ*M H_2_-DCFDA in Hank’s balanced salt solution (HBSS) for 20 min at 37 °C in darkness. Then the cells were collected, centrifuged at 120×*g* for 5 min, resuspended in PBS and analysed using an Epics XL flow cytometer with excitation and emission settings at 480 and 525 nm, respectively. Intracellular ROS were also directly visualized by means of a fluorescence microscope.^31^ In this case, cells were seeded in 96-well plates (8×10^3^/well) and after 24 h were stimulated with PN for 1 h, washed with PBS and incubated in 100 *μ*l of 10 *μ*M H_2_-DCFDA in HBSS for 20 min at 37 °C in darkness. Finally, H_2_-DCFDA was replaced with PBS and after other 20 min fluorescence produced by oxidation of fluorochrome was visualized with a Leica microscope equipped with a DC300 F camera at ×200 magnification using FITC filter (excitation wavelength of 485 nm and emission wavelength of 530 nm).

### Transient gene silencing by siRNA

siRNAs against Nrf2 (Nrf2 siRNA) and scrambled siRNA (control siRNA), as a negative non-silencing control, were purchased from Santa Cruz Biotechnology (Santa Cruz, CA, USA).

Cells (10^5^/well) were seeded in six-well plates and cultured in antibiotic and serum-free DMEM supplemented with 2.0 mM glutamine and 1% non-essential amino acids, until ~50% confluence. Then, cells were transfected with 50 nM siNrf2 in the presence of 5 *μ*l Lipofectamine 2000 (Invitrogen Life Technologies, Monza, Italy) in a final volume of 1 ml serum-free medium.^67^ The reaction was stopped after 5 h replacing the culture medium with fresh DMEM + 10% FBS. After 24 h from transfection, silenced cells were employed for the experiments.

### Western blotting analysis

Cell lysates and protein samples were prepared as previously reported.^67^ Equal amounts of protein samples (20 *μ*g per lane) were run in a SDS-polyacrylamide gel electrophoresis and then transferred to a nitrocellulose membrane. All analyses were performed using specific primary antibodies against: Bcl-2, Nrf2, MnSOD and P-gp, which were supplied from Santa Cruz Biotechnology; HSP70 and NQO1 from Cell Signaling Technology (Beverly, MA, USA); and catalase and *β*-actin from Sigma-Aldrich. Then, the detection was developed by using a secondary antibody conjugated with horseradish peroxidase (Santa Cruz Biotechnology). The protein bands were revealed with an enhanced chemiluminescence detection system (Bio-Rad, Hercules, CA, USA) and visualized by ChemiDoc XRS system (Bio-Rad) and Quality One 4.5.2 software (Bio-Rad). The intensity of the protein bands was quantified by densitometric analysis using SMX Image software (Bio-Rad). The correct protein loading was ascertained by red Ponceau staining and immunoblotting for *β*-actin. All the blots shown are representative of at least four different experiments.

### Statistical analysis

Results are presented as means±S.D. of data obtained from at least three independent experiments. Data were analysed using Student’s *t*-test. A *P*-value below 0.01 was considered significant.

## Additional information

**Publisher’s note:** Springer Nature remains neutral with regard to jurisdictional claims in published maps and institutional affiliations.

## Figures and Tables

**Table 1:** Knockdown of Nrf2 prevented in MDA-MB231 cells the development of resistance induced by Mitox

*Dose–response curve towards:*	*IC*_*50*_ *values (μM)*				*RI values*	
	*Control siRNA*		*Nrf2 siRNA*		*Control siRNA (b/a)*	*Nrf2 siRNA (b/a)*
	*a*	*b*	*a*	*b*		
PN	5.2±0.50	16.2±1.5	5.0±0.40	9.0±0.95	3.1±0.33	1.8±0.20
Mitox	0.7±0.08	1.4±0.15	0.9±0.08	1.0±0.10	2.0±0.20	1.1±0.12
DOX	0.8±0.08	1.6±0.16	0.9±0.08	1.2±0.12	2.0±0.22	1.3±0.15

Abbreviations: a, parental cells; b, mitox-resistant cells; DOX, doxorubicin; Mitox, mitoxantrone; Nrf2, NF-E2-related factor 2; PN, parthenolide; RI, resistance index; siRNA, small interference RNA.

Parental MDA-MB231 cells were transfected with Nrf2 siRNA or with control siRNA, as reported in Materials and Methods. After 24 h, the cells were submitted for 7 days to treatment with 10 nM Mitox in order to induce resistance. At the end, the cells were collected, counted and employed to test, in comparison with untreated cells, the effect exerted on cell viability by various doses of PN, Mitox and DOX. Dose–response curves (not shown) permitted to calculate IC_50_ and RI values reported in the Table. Results are the means±S.D. of three different experiments.
